# Editorial: The genetics of inherited retinal diseases in understudied ethnic groups: Novel associations, challenges, and perspectives

**DOI:** 10.3389/fgene.2022.990782

**Published:** 2022-08-23

**Authors:** Said El Shamieh, Paolo Enrico Maltese

**Affiliations:** ^1^ Department of Medical Laboratory Technology, Faculty of Health Sciences, Beirut Arab University, Beirut, Lebanon; ^2^ Magi’s Lab SRL, Rovereto, Italy

**Keywords:** inherited retinal degenerations, understudied ethnic groups, mutations, gene-disease association, next generation sequencing

Inherited retinal dystrophies (IRDs) cover a broad array of rare retinal diseases with diversify in genetic bases and phenotypes, affecting roughly as many as one in 2,000 individuals (Chen et al., 2021). Night or color blindness, tunnel vision, and later development to total blindness are all common vision impairment manifestations of IRDs that usually worsen with age (Chen et al., 2021). Cases of IRDs may be syndromic if they are coupled with extra-ocular symptoms or non-syndromic if they are restricted to the eye (Hartong, 2006; Berson, 2006; Dryja, 2006). Interestingly, IRDs include over 20 different phenotypes (Hartong et al., 2006). The age of onset, the rate of progression, and the underlying causal genes may assist in categorizing these distinct phenotypes (Hartong et al., 2006; Perea-Romero et al., 2021). Retinitis pigmentosa (RP) (OMIM 500004), also known as rod-cone dystrophy, is the most common non-syndromic IRD, affecting around 1.5 million individuals globally (Chen et al., 2021). Other non-syndromic forms include cone-rod dystrophies (OMIM 120970), Stargardt disease (OMIM 248200), X-linked retinoschisis (OMIM 312700) and many others. On the other hand, the most common syndromic form is Usher syndrome (USH) (OMIM 276900), a combination of hearing loss and RP (Castiglione and Moller, 2022).

Although several genetic studies have identified novel IRDs-associated genes and genetic variations, most of these associations and gene prevalence data were based on cohorts in Western Europe and North America (Smirnov et al., 2021; Colombo et al., 2021). Although the genetic basis of IRDs varies among patient cohorts, even replication for the major findings is still lacking in understudied ethnicities (Jaffal et al., 2021). Growing evidence is continuously showing the importance of better investigating these ethnicities (Amish, Mennonites, Turks, Middle Eastern populations, and the Ashkenazi Jews) because their structure and history of consanguinity are the most valuable and amenable for genomics studies. Therefore, we aimed in this Research Topic to put some of these ethnicities under the loop by including five contributions to the IRD field from different cohorts around the globe.

Maltese et al. reported the clinical and genetic features of an ethnically heterogeneous group of 123 IRD patients from different underrepresented countries referred to specialized Italian centers. These affected individuals were from all over the world; 27% were North/Northwest Africans, 17% were Asians, 15% were South/Central Americans, and 41% were Eastern Europeans. Whereas RP showed the highest prevalence at the phenotype level (56%), *ABCA4* (OMIM 601691) mutations were the most frequent at the genotype level. Among the 113 different variants identified, 33 were unpublished and were associated with IRDs for the first time in this study. This work described the clinical and genetic characteristics of IRD patients from understudied ethnic groups in Italy and expanded the epidemiological research on understudied ethnic groups.

Jaffal et al., 2022a searched for the causative mutations in nine Lebanese families with USH using whole-exome sequencing. This study identified four novel disease-causing mutations in *USH2A* (OMIM 608400)*, ADGRV1* (OMIM 602851)*, PCDH15* (OMIM 601067) and *CDH23* (OMIM 601067) genes, respectively. Moreover, a meta-analysis conducted by these authors showed that the frequency of USH type 3 had a relatively high incidence (23%) in Lebanon compared to the worldwide prevalence and that, till today, the major USH genes in the Lebanese populations are *ADGRV1*, *USH2A*, and *CLRN1* (OMIM 606397) since they are responsible for around 75% of the cases Jaffal et al., 2022a The study broadened the spectrum of USH-causing mutations also showing a high heterogeneity of this disease in Lebanon.

Guo et al. performed a detailed phenotypic analysis of the *Retinoschisis 1* (*RS1*) (OMIM 300839) mutations associated with X-linked juvenile retinoschisis (XLRS) in ten Chinese families. In addition to identifying a novel mutation in *RS1*; c.657C>A; p. (Cys219*), the authors noticed a high degree of phenotypic heterogeneity; asymmetrical fundus manifestations were noticed in some cases. Importantly, their approach allowed diagnosing three patients not showing signs of macular retinoschisis Guo et al. Therefore, authors concluded that genetic testing is highly recommended in patients with asymmetric fundus appearance, macular atrophy, cystic degeneration and amblyopia, for making a precise diagnosis.

Kannabiran et al. described the studies published concerning RP’s genetics in India and neighboring South Asian countries like Pakistan. There is indeed a significant opportunity for gene discovery in India and South Asia, where there is not much known about IRDs compared to the number of affected individuals Kannabiran et al. *RP32* (OMIM 609913) locus was also mapped in a consanguineous Pakistani family with autosomal recessive RP, and the subsequent sequencing and refining led to the identification of the c.75C>A; p. (Asp25Glu) in Chloride channel CLIC like 1 (*CLCC1*) (OMIM 617539) (Li et al., 2017). The c.75C>A is the only reported mutation in *CLCC1* to date and accounts for around 6% of genetic cases of arRP in Pakistani families (Li et al., 2017). Ma et al. hypothesized that this mutation arose from a common founder and thus studied all the identified patients with this mutation (Li et al., 2017). By studying the surrounding haplotypes, the authors estimated that this mutation arose 2,000–5,000 years ago and has been transmitted to affected families dispersed worldwide (Li et al., 2017). *RP25* (OMIM 602772), *RP32*, and other loci reported so far reveal the potential for novel gene discovery in the understudied ethnicities and for improving our understanding of the IRDs genetics. Although exome sequencing is the gold standard methodology to identify novel disease-associated genes, the authors highlighted that investigating populations with a high degree of consanguinity and inbreeding might help the scope regardless of the employed method.

In conclusion, the findings of this Research Topic highlight the usefulness of focusing on understudied ethnic groups. Such a focus represents an opportunity to strengthen our knowledge of IRDs genetics, improve the diagnostic yield of genetic testing, and might lead to new treatments. To go further, scientists working on understudied ethnicities need to implement NGS, undergo international collaborations, and improve the accessibility of their patients to the healthcare service (Jaffal et al., 2022b).
